# The Mitochondrial Genome of *Elodia flavipalpis* Aldrich (Diptera: Tachinidae) and the Evolutionary Timescale of Tachinid Flies

**DOI:** 10.1371/journal.pone.0061814

**Published:** 2013-04-23

**Authors:** Zhe Zhao, Tian-juan Su, Douglas Chesters, Shi-di Wang, Simon Y. W. Ho, Chao-dong Zhu, Xiao-lin Chen, Chun-tian Zhang

**Affiliations:** 1 Liaoning Key Laboratory of Evolution and Biodiversity, Shenyang Normal University, Shenyang, Liaoning, China; 2 Key Laboratory of Zoological Systematics and Evolution, Institute of Zoology, Chinese Academy of Sciences, Beijing, China; 3 School of Biological Sciences, University of Sydney, Sydney, New South Wales, Australia; Natural Resources Canada, Canada

## Abstract

Tachinid flies are natural enemies of many lepidopteran and coleopteran pests of forests, crops, and fruit trees. In order to address the lack of genetic data in this economically important group, we sequenced the complete mitochondrial genome of the Palaearctic tachinid fly *Elodia flavipalpis* Aldrich, 1933. Usually found in Northern China and Japan, this species is one of the primary natural enemies of the leaf-roller moths (Tortricidae), which are major pests of various fruit trees. The 14,932-bp mitochondrial genome was typical of Diptera, with 13 protein-coding genes, 22 tRNA genes, and 2 rRNA genes. However, its control region is only 105 bp in length, which is the shortest found so far in flies. In order to estimate dipteran evolutionary relationships, we conducted a phylogenetic analysis of 58 mitochondrial genomes from 23 families. Maximum-likelihood and Bayesian methods supported the monophyly of both Tachinidae and superfamily Oestroidea. Within the subsection Calyptratae, Muscidae was inferred as the sister group to Oestroidea. Within Oestroidea, Calliphoridae and Sarcophagidae formed a sister clade to Oestridae and Tachinidae. Using a Bayesian relaxed clock calibrated with fossil data, we estimated that Tachinidae originated in the middle Eocene.

## Introduction

Since the first insect mitochondrial genome (mitogenome) sequence was reported by Clary and Wolstenholme in 1985 [1], Diptera has remained the primary model system for mitogenomic research. This has included such diverse topics as species identification [2], [3], molecular evolution and phylogenetic inference [4]–[7], population structure and phylogeography [8]–[12], and genome structure and rearrangement [13]–[19]. Because of its small size and relative ease of sequencing, the number of mitogenome sequences has grown rapidly. As of January 2013, there are 64 complete or near-complete dipteran mitogenome sequences in GenBank, accounting for about 17.5% of the 365 insect mitogenomes that have been sequenced. In addition to the model organism *Drosophila*, most studies of Diptera have focused on taxa of medical and economic importance, such as the anopheline mosquitoes (Culicidae), which are vectors of malaria [20], [21]; the fruit flies *Ceratitis capitata* and *Bactrocera* spp. (Tephritidae), which are serious agricultural pests [11], [22]; the blowflies (Calliphoridae) and oestrid flies (Oestridae), which can cause myiasis [23], [24]; and leaf-miners (Agromyzidae), which are vegetable and horticultural pests [16], [25].

Tachinid flies have a worldwide distribution and comprise nearly 10,000 described species [29]. Despite Tachinidae being the second-largest dipteran family, the mitogenomes of only two species have been sequenced completely: *Exorista sorbillans* (Exoristinae, Exoristini) and *Rutilia goerlingiana* (Dexiinae; Rutiliini) [26], [27], [28]. They are natural enemies of many lepidopteran and coleopteran pests of forests, agricultural crops, and fruit trees, and thus are of economic importance. The Palaearctic tachinid fly, *Elodia flavipalpis* Aldrich, 1933 (Exoristinae, Goniini), is usually found in Northern China and Japan and is in the same subfamily, Exoristinae, as *Ex. sorbillans* [30], [31]. It is one of the primary natural enemies of the leaf-roller moths (Tortricidae), which are major pests of various fruit trees [32], [33]. The monophyly of Tachinidae is broadly supported by phylogenetic studies, but questions remain about its place in the superfamily Oestroidea, particularly the relationship between Tachinidae and several large families of Oestroidea [27], [28], [34]–[36]. There have been various studies of the divergence times of different groups of flies [7], [11], [12], [37], with a recent study placing the rapid radiation of Schizophora 65 mya in the Paleocene [28]. However, owing to the uncertain taxonomic position of Tachinidae in Oestroidea, the evolutionary timescale of tachinid flies has not been well studied.

Here we describe the complete mitochondrial genome of *El. flavipalpis*. The mitogenome contributes to discussion on the evolutionary relationships and taxonomic positions of the Tachinidae, puts forward a bold hypothesis regarding the timescale in which the family originated, and will aid further molecular research of related taxa through the findings on primer selection for atypical regions and the optimization of PCR experiments.

## Materials and Methods

### Specimen Collection, DNA Extraction, and DNA Amplification

Adult individuals of *Elodia flavipalpis* Aldrich, 1933 were collected directly from the pupae of their host species, the leaf-roller moth *Spilonota lechriaspis* Meyrick (Tortricidae). Moth pupae were collected at an organic apple orchard in Beijing, China, and hatched in the laboratory. The specimens were preserved in 99.5% ethanol and stored at −20°C for preservation of nucleic acids. DNA extraction from a single specimen was performed using the DNeasy Tissue kit (QIAGEN) following the manufacturer’s instructions.

The fragments were first amplified with the universal PCR primers from Simon et al. [38] and some dipteran-specific primers from Han [39] and Weigl et al. [24] (Table 1). Primer pairs for amplification of the mitochondrial control region were modified according to Lessinger et al. [40] and Oliveira et al. [41]. Species-specific primers were designed using Primer Premier 6.0 software [42], based on the initial fragments aligned with sequences from three closely related species, *Ex. sorbillans*, Calliphoridae spp., and Oestridae spp. (Table 2). PCR products covering the remaining regions of the mitogenome were amplified using universal and species-specific primers (Table 1). The entire genome of *El. flavipalpis* was amplified in 21 fragments. All of the primers were synthesized by Shanghai Sangon Bio-technology Co., Ltd (Beijing, China).

**Table 1:** Details of mitogenome sequencing protocols used in this study.

Region	Primer pairs (F/R)	Sequence (forward and reverse) 5′→3′	Size (bp)	PCR conditions (annealing/extension)
*ND2*	TM-J-206^a/^N2-N-732^a^	GCTAA-ATAAAGCTAACAGGTTCAT/GAAGTTTGGTTTAAACCTCC	∼500	49°C, 45 s/72°C, 1 min
*ND2*-*CO1*	N2-J-283^d/^C1-N-1740^d^	CATGACTAGGAACTTGAATAGG/AGAACTAAAGCAGGAGGTAA	∼1500	47°C, 45 s/68°C, 4 min
*CO1*	TY-J-1460^a/^C1-N-2191^a^	TACAATTTATCGCCTAAA-CTTCAGCC/CCCGGTAAAATTAAAATATAAACTTC	∼700	49°C, 45 s/72°C, 1 min
*CO1*	C1-J-1751^a/^TL2-N-3014^a^	GGATCACCTGATATAGCATTCCC/TCCAATGCACTAATCTGCCATATTA	∼1200	52°C, 30 s/72°C, 1 min
*CO1*-*CO3*	Cl-J-2183^a/^C3-N-5460^a^	CAACATTTATTTTGATTTTTTGG/TCAACAAAGTGTCAGTATCA	∼3200	44.5°C, 45 s/68°C, 5 min
*ATP8*	C2-J-3530^d^/A6-N-4493^d^	AAGTTGATGGAACTCCTGGA/GTAAGTCGAACTGCTAATGT	∼950	49°C, 45 s/72°C, 1 min, 50 s
*CO3*-*ND3*	C3-J-5005^d/^E-rev^b^	CTCCATCAGTTGAATTAGGTGCTA/AGTGATAAGCCTCTTTTTGGCTTC	∼1050	49°C, 45 s/72°C, 1 min, 50 s
*ND5*	F-fw^b/^N5-N-7707^d^	CATTTGATTTGCATTCAAAAAGTATTG/AGGATGAGATGGATTAGGAT	∼1800	45°C, 45 s/68°C, 3 min
*ND5*-*ND4*	H-fw^b/^N4-N-8718^a^	GAAACAGGAGTAGGAGCTGCTATAGC/GCTTATTCATCGGTTGCTCA	∼1200	53°C, 45 s/72°C, 1 min, 50 s
*ND4*	I-fw^b/^N4-N-8924^a^	CAATTCTATTAATTAAAGAAATTTCTCC/AAAGCTCATGTTGAAGCTCC	∼550	48°C, 45 s/72°C, 1 min, 50 s
*ND4*	N4-J-8614^d/^N4-N-9061^d^	TGAGCAACAGAAGAATAAGC/ATCAACCAGAACGATTACAAG	∼400	47°C, 45 s/72°C, 1 min, 50 s
*ND4*-*ND6*	N4-J-8944^a/^I-rev^b^	GGAGCTTCAACATGAGCTTT/CTTATTTTTGATTTACAAGACCAATG	∼850	49°C, 45 s/72°C, 1 min, 20 s
*ND4*- *CYTB*	N4-J-9511^d/^CB-N-11218^d^	CTAAAATTGATAACCCTAAAGC/TCAGGTTGAATGTGAATTGG	∼1700	45°C, 45 s/68°C, 3 min
*CYTB*-*ND1*	CB-J-10933^a/^N1-N-12051^a^	TATGTACTACCATGAGGACAAATATC/GATTTTGCTGAAGGTGAATCAGA	∼1100	50.5°C, 45 s/72°C, 1 min, 50 s
*ND1*- lrRNA	N1-J-11891^d/^16S-N-12855^d^	ATCCTCCTCTTCTATATTCAAT/GATTGCGACCTCGATGTT	∼950	48°C, 45 s/72°C, 1 min, 30 s
lrRNA	LR-J-12883^c/^LR-N-13398^a^	CTCCGGTTTGAACTCAGATC/CGCCTGTTTATCAAAAACAT	∼550	47°C, 45 s/72°C, 2 min
lrRNA - srRNA	LR-J-12888^d/^SR -N-14373^d^	ACGCTGTTATCCCTAAAGTA/AATCCACGATGAACCTTACT	∼1500	45°C, 45 s/68°C, 3 min
srRNA	SR-J-14233^a/^SR-N-14756^a^	AAGAGCGACGGGCGATGTGT/GACAAA-ATTCGT-GCCAGCAGT	∼500	50°C, 45 s/72°C, 1 min, 30 s
srRNA	SR-J-14612^a/^SR-N-14922^a^	AGGGTATCTAATCCTAGTTT/AAGTTTTATT-TTGGCTTA	∼300	43°C, 45 s/72°C, 1 min
srRNA-*ND2*	SR-J-14646^e^/N2-N-757^d^	GCTGGCACAAATTAAATC/GCTGCAAGTATTCAACTTAAATG	∼1150	43°C, 1 min/68°C, 6 min
Control region	SR-J-14646^e/^N2-N-309^f^	GCTGGCACAAATTAAATC/CTAAACCTATTCAAGTTCC	∼650	42°C, 1 min/68°C, 6 min

Note: *CO1*, *CO2*, *CO3*: cytochrome c oxidase subunit 1, 2, and 3 genes; *CYTB*: cytochrome b gene; *ATP6*, *ATP8*: ATP synthase subunit 6 and 8 genes; *ND1*, *ND2*, *ND3*, *ND4*, *ND4L*, *ND5*, *ND6*: NADH dehydrogenase subunit 1–6 and 4L genes. *lrRNA, srRNA*: large and small ribosomal RNA.

a Primers from Simon et al. [27].

b Primers from Weigl et al. [21].

c Primers from Han [28].

d Primers designed specially for this genome, using the nomenclature of Simon et al. [27].

e Primers modified from Lessinger et al. [29]. f Primers modified from Oliveira et al. [30].

**Table 2:** Summary of mitogenome sequences from Diptera and an outgroup species from Lepidoptera.

Species	Family	Length (bp)	Accession Number	Reference
*Elodia flavipalpis*	Tachinidae	14932	NC_018118	Present study
*Exorista sorbillans*	Tachinidae	14960	NC_014704	Shao et al., 2012
*Dermatobia hominis*	Oestridae	16360	NC_006378	Azeredo-Espin et al.,2004
*Hypoderma lineatum*	Oestridae	16354	NC_013932	Weigl et al., 2010
*Chrysomya putoria*	Calliphoridae	15837	NC_002697	Junqueira et al., 2004
*Cochliomyia hominivorax*	Calliphoridae	16022	NC_002660	Lessinger et al., 2000
*Lucilia sericata*	Calliphoridae	15939	NC_009733	Stevens et al., 2008
*Sarcophaga impatiens*	Sarcophagidae	15169	NC_017605	Nelson et al., 2012
*Haematobia irritans*	Muscidae	16078	NC_007102	Oliveira et al., 2008
*Drosophila littoralis*	Drosophilidae	16017	NC_011596	Sorokina et al., 2010
*Drosophila sechellia*	Drosophilidae	14950	NC_005780	Ballard, 2000 a
*Drosophila simulans*	Drosophilidae	14927	NC_005781	Ballard, 2000 a
*Drosophila mauritiana*	Drosophilidae	14964	NC_005779	Ballard, 2000 b
*Drosophila melanogaster*	Drosophilidae	19517	NC_001709	Lewis et al., 1995
*Drosophila yakuba*	Drosophilidae	16019	NC_001322	Clary & Woolstenholme, 1985
*Drosophila pseudoobscura*	Drosophilidae	14914	NC_018348	Torres et al., 2009
*Liriomyza sativae*	Agromyzidae	15551	NC_015926	Yang et al., 2011
*Liriomyza trifolii*	Agromyzidae	16141	NC_014283	Wang, 2010
*Liriomyza bryoniae*	Agromyzidae	16183	NC_016713	Yang et al. unpublished
*Liriomyza huidobrensis*	Agromyzidae	16236	NC_016716	Yang et al. unpublished
*Fergusonina taylori*	Fergusoninidae	16000	NC_016865	Nelson et al., 2011
*Ceratitis capitata*	Tephritidae	15980	NC_000857	Spanos et al. 2000
*Bactrocera carambolae*	Tephritidae	15915	NC_009772	Ye et al., 2010
*Bactrocera dorsalis*	Tephritidae	15915	NC_008748	Yu et al., 2007
*Bactrocera minax*	Tephritidae	16043	NC_014402	Zhang et al.,2010
*Bactrocera oleae*	Tephritidae	15815	NC_005333	Nardi et al.,2003
*Bactrocera papayae*	Tephritidae	15915	NC_009770	Ye et al., 2010
*Bactrocera philippinensis*	Tephritidae	15915	NC_009771	Ye et al.,2010
*Bactrocera tryoni*	Tephritidae	15925	NC_014611	Nardi et al.,2010
*Bactrocera cucurbitae*	Tephritidae	15825	NC_016056	Wu,P.-F. unpublished
*Simosyrphus grandicornis*	Syrphidae	16141	NC_008754	Cameron et al., 2007
*Trichophthalma punctata*	Nemestrinidae	16396	NC_008755	Cameron et al., 2007
*Cydistomyia duplonotata*	Tabanidae	16247	NC_008756	Cameron et al., 2007
*Rhopalomyia pomum*	Cecidomyiidae	14503	NC_013063	Beckenbach and Joy, 2009
*Mayetiola destructor*	Cecidomyiidae	14759	NC_013066	Beckenbach and Joy, 2009
*Culicoides arakawae*	Ceratopogonidae	18132	NC_009809	Matsumoto et al.,2009
*Aedes aegypti*	Culicidae	16655	NC_010241	Behura et al., 2011
*Aedes albopictus* ^a^	Culicidae	16665	NC_006817	Ho, C.-M. et al. unpublished
*Anopheles darlingi*	Culicidae	15386	NC_014275	Moreno et al., 2010
*Anopheles funestus^b^*	Culicidae	–	NC_008070	Krzywinski et al., 2006
*Anopheles gambiae*	Culicidae	15363	NC_002084	Beard et al., 1993
*Anopheles quadrimaculatus*	Culicidae	15455	NC_000875	Mitchell et al., 1993
*Anopheles albitarsis*	Culicidae	15413	HQ_335344	Krzywinski et al., 2011
*Anopheles deaneorum*	Culicidae	15424	HQ_335347	Krzywinski et al., 2011
*Anopheles janconnae*	Culicidae	15425	HQ_335348	Krzywinski et al., 2011
*Anopheles oryzalimnetes*	Culicidae	15422	HQ_335345	Krzywinski et al., 2011
*Culex pipiens*	Culicidae	14856	NC_015079	Atyame et al., 2011
*Culex quinquefasciatus*	Culicidae	15587	NC_014574	Behura et al., 2011
*Chironomus tepperi*	Chironomidae	15652	NC_016167	Beckenbach, 2012
*Trichocera bimacula*	Trichoceridae	16140	NC_016169	Beckenbach, 2012
*Paracladura trichoptera*	Trichoceridae	16143	NC_016173	Beckenbach, 2012
*Sylvicola fenestralis*	Anisopodidae	16234	NC_016176	Beckenbach, 2012
*Bittacomorphella fenderiana*	Ptychopteridae	15609	JN_861745	Beckenbach, 2012
*Ptychoptera sp.*	Ptychopteridae	15214	NC_016201	Beckenbach, 2012
*Protoplasa fitchii*	Tanyderidae	16154	NC_016202	Beckenbach, 2012
*Cramptonomyia spenceri*	Pachyneuridae	16274	NC_016203	Beckenbach, 2012
*Arachnocampa flava*	Keroplatidae	16923	NC_016204	Beckenbach, 2012
*Tipula abdominalis*	Tipulidae	14566	JN_861743	Beckenbach, 2012
*Spilonota lechriaspis*	Tortricidae (Lepidoptera)	15368	NC_014294	Zhao et al., 2010

Note ‘−’ not available (unknown or incomplete data).

In order to reduce time required for sequencing and walking, we used both standard and nested PCR techniques. The PCR conditions for all of the fragments are shown in Table 1. The control region was amplified using a nested PCR approach. The “external” primers SR-J-14646 and N2-N-757 were used for the first step of the PCR (long target), followed by nested amplification (specific target) with the “internal” primers SR-J-14646 and N2-N-309. All of the fragments were amplified using TaKaRa LA *Taq* (Takara Co., Dalian, China), and performed on an Eppendorf Mastercycler gradient in 50 µl reaction volumes. The reaction volume consisted of 25.5 µl of sterilized distilled water, 5 µl of 10× LA PCR Buffer II (Takara), 5 µl of 25 mM MgCl2, 8 µl of dNTPs Mixture, 1.5 µl of each primer (10 µM), 3 µl of DNA template, and 0.5 µl (1.25 U) of TaKaRa LA *Taq* polymerase (Takara).

### Sequence Assembly and Annotation

The PCR products were detected via electrophoresis in 1% agarose gel, purified using the 3S Spin PCR Product Purification Kit, and sequenced directly with the ABI-377 automatic DNA sequencer. All amplified products were sequenced directly using upstream and downstream primers from both directions. Sequencing was performed using ABI BigDye ver 3.1 dye-terminator sequencing technology and run on ABI PRISM 3730 × 1 capillary sequencers. Raw sequences were manually checked and assembled using the software BioEdit version 7.0.9.0 [43] and SeqMan (DNAStar, Steve ShearDown, 1998–2001 version reserved by DNASTAR Inc., Madison, Wisconsin, USA).

Protein-coding and ribosomal RNA genes and gene boundaries were identified by BLAST search (http://www.ncbi.nlm.nih.gov/BLAST) and by comparison with sequences from other brachyceran species. The genomic positions and secondary structures of 20 of the 22 transfer RNAs (tRNAs) were identified by tRNAscan-SE software v.1.21 [44], with the remaining two (tRNA^Arg^ and tRNA^Ser(AGN)^) identified by visual inspection of genome sequence followed by inference using RNAstructure Ver.5.3 [45] (Table 3). Nucleotide composition was calculated using MEGA 4.1 [46]. Sequence data have been deposited in the NCBI database [GenBank: NC_018118].

**Table 3:** Organization of the mitogenome of *Elodia flavipalpis* Aldrich.

Gene	Strand	Location^a^		Length(bp)	IGN^b^	CodonStart/Anti Stop		AT%
tRNA^Ile^	+		1–65	65	−3	GAT		77.0
tRNA^Gln^	−		63–131	69	6	TTG		86.2
tRNA^Met^	+		138–206	69	0	CAT		71.0
*ND2*	+		207–1217	1011	−2	ATT	TAA	83.4
tRNA^Trp^	+		1216–1283	68	−8	TCA		80.9
tRNA^Cys^	−		1276–1342	67	2	GCA		74.6
tRNA^Tyr^	−		1344–1408	65	6	GTA		80.0
*CO1*	+		1415–2953	1539	−5	TCG	TAA	72.9
tRNA^Leu(UUR)^	+		2949–3014	66	4	TAA		77.3
*CO2*	+		3019–3706	688	0	ATG	T	77.0
tRNA^Lys^	+		3707–3777	71	−1	CTT		69.0
tRNA^Asp^	+		3777–3847	71	0	GTC		87.4
*ATP8*	+		3848–4012	165	−7	ATT	TAA	86.8
*ATP6*	+		4006–4683	678	−1	ATG	TAA	78.4
*CO3*	+		4683–5471	789	6	ATG	TAA	73.0
tRNA^Gly^	+		5478–5542	65	0	TCC		83.1
*ND3*	+		5543–5896	354	0	ATT	TAA	82.2
tRNA^Ala^	+		5897–5963	67	0	TGC		79.1
tRNA^Arg^	+		5966–6027	62	11	TCG		72.6
tRNA^Asn^	+		6039–6103	65	0	GTT		78.5
tRNA^Ser(AGN)^	+		6104–6171	68	0	GCT		75.0
tRNA^Glu^	+		6172–6233	62	18	TTC		92.0
tRNA^Phe^	−		6252–6316	65	0	GAA		80.0
*ND5*	−		6317–8036	1720	15	ATT	T	80.9
tRNA^His^	−		8052–8115	64	1	GTG		84.4
*ND4*	−		8117–9455	1339	−7	ATG	T	81.4
*ND4L*	−		9449–9745	297	2	ATG	TAA	84.5
tRNA^Thr^	+		9748–9812	65	0	TGT		86.1
tRNA^Pro^	−		9813–9877	65	2	TGG		81.5
*ND6*	+		9880–10404	525	−1	ATT	TAA	86.7
*CYTB*	+		10404–11540	1137	−2	ATG	TAG	75.9
tRNA^Ser(UCN)^	+		11539–11605	67	16	TGA		82.1
*ND1*	−		11622–12569	948	1	TTG	TAA	81.1
tRNA^Leu(CUN)^	−		12571–12635	65	0	TAG		81.6
lrRNA	−		12636–13972	1337	0			84.6
tRNA^Val^	−		13973–14044	72	0	TAC		80.6
srRNA	−		14045–14827	783	0			81.7
Control region	−		14828–14932	105	0			92.4

Note:

a Gene positions with parentheses indicate the genes encoded by major strand; plus (+) and minus (−) symbols represent major and minor strands, respectively.

b IGN: Intergenic nucleotide, minus indicates overlapping between genes. tRNA*^X^*: where X is the abbreviation of the corresponding amino acid.

### Phylogenetic Analysis

We conducted a phylogenetic analysis using all of the mitogenomes of Diptera that were available as of August 2012 (58 species from 33 genera and 23 families), along with a lepidopteran outgroup species (*Spilonota lechriaspis*) (Table 2). To assess the impact of saturation at third codon positions, we constructed two mitochondrial sequence alignments. The first dataset consisted of all 13 protein-coding genes and the 2 ribosomal RNA genes. The second dataset was the same, except for the exclusion of the third codon sites from the protein-coding genes. Sequences of protein-coding genes were translated into amino acid sequences, aligned using ClustalX v2.0.9 [47], then back-translated. Ribosomal RNA genes were also aligned using ClustalX, and refined by eye to account for their secondary structures. The GTR+I+G model was selected by the Akaike information criterion for both datasets in MODELTEST v.3.7 [48]. Maximum likelihood (ML) and Bayesian methods were used for phylogenetic analysis. For the ML analyses, we used RAxML v7.0.4 [49] with 1000 bootstrap replicates. The Bayesian phylogenetic analysis was performed using MrBayes v3.1.2. [50], with posterior distributions estimated using Markov chain Monte Carlo (MCMC) sampling. We conducted two independent runs, each with one cold and three heated chains. Samples were drawn every 100 MCMC steps over a total of 2 million steps. The first 25% of steps were discarded as burn-in.

Divergence times were estimated using the Bayesian phylogenetic method implemented in BEAST v.1.6.2 [51]. Rate variation among lineages was modelled using an uncorrelated lognormal relaxed clock [52], with the tree prior generated using a Yule speciation model. Based on previous research on divergence times in Diptera [7], [11], [12], [28], [37], we implemented fossil-based minimum ages for three clades and added one maximum age constraint. The origin of Diptera was assumed to be between 270 and 230 million years ago (mya) [28], [53]. We also specified minimum age constraints of 195 mya for Brachycera and 64 mya for Schizophora [53]–[55]. Posterior estimates of parameters were obtained by MCMC sampling. Samples were drawn every 1000 steps over a total of 10 million steps. Tracer v1.5 [56] was used to confirm that the effective sample size of each parameter exceeded 100. The maximum-clade-credibility tree was calculated using TreeAnnotator v1.6.2, with the node times scaled to match mean posterior estimates.

## Results and Discussion

### Genome Organization

**Figure 1:**
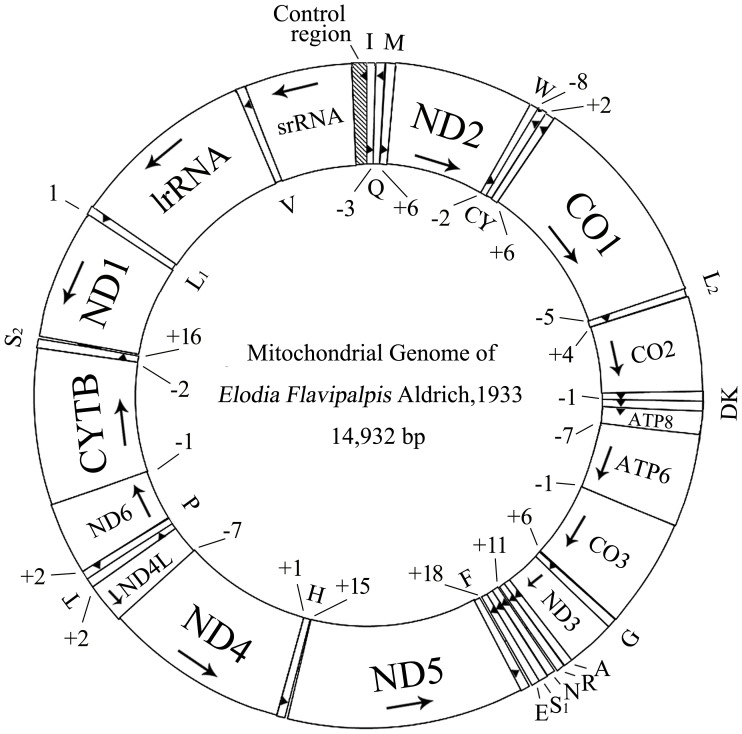
Mitochondrial genome map of *Elodia flavipalpis*. Numbers indicate non-coding nucleotides between genes (positive values) or gene overlap (negative values). Arrows indicate orientation on (+) strand (clockwise) or (−) strand (counterclockwise).

The 14932 bp mitogenome of *El. flavipalpis* contained all 37 genes usually present in bilaterians: 13 protein-coding genes, 22 tRNA genes, and 2 rRNA genes (Figure 1, Table 3). The gene order corresponds to the typical pattern of brachyceran flies, such as *Drosophila* spp. [1], [22], [57]. Gene order is typically conserved in brachyceran flies, except for a few species in which additional tRNAs have been detected in the control region (*Liriomyza trifolii*, *Chrysomya* spp., and *Cydistomyia duplonotata*), and *Dermatobia hominis*, in which an additional tRNA^Val^ is found on the minor strand. In contrast, there is an elevated number of tRNA rearrangements in nematoceran flies [13], [14], [20], [21].

In total, there were 37 overlapping nucleotides between neighboring genes at 10 locations, with the length of overlapping sequence ranging from 1 to 7 bp. Excluding the control region, there were 90 intergenic nucleotides at 12 locations, in stretches ranging from 1 to 18 bp (Table 3).

As with other insects, the nucleotide composition of the *El. flavipalpis* mitogenome was biased towards A and T. The overall A+T content was 79.97%, slightly higher than that of *Ex. sorbillans* (78.4%) and *R. goerlingiana* (77.7%), and is among the highest of the sequenced dipteran species (67.2%–85.2%; Table S1). A detailed comparison of nucleotide composition, AT-skew, and GC-skew between the two closely related species, *El. flavipalpis*, *Ex. sorbillans* and *R. goerlingiana*, is given in Table 4.

**Table 4:** Comparison of mitochondrial nucleotide composition in three tachinid flies.

Region	A+T %			G+C %			AT-skew			GC-skew		
	*El. fla*	*Ex. sor*	*R. goe*	*El. fla*	*Ex. sor*	*R. goe*	*El. fla*	*Ex. sor*	*R. goe*	*El. fla*	*Ex. sor*	*R. goe*
Whole mitogenome	79.9	78.4	77.7	20.1	21.5	22.3	0.00	0.02	0.04	−0.15	−0.17	−0.23
Protein-coding genes	79.1	77.7	76.2	20.9	22.3	23.8	−0.14	−0.15	−0.15	0.04	0.00	0.00
1st codon position	71.2	70.4	70.1	28.8	29.6	29.9	−0.19	−0.18	−0.07	0.12	0.09	0.22
2nd codon position	79.1	78.0	67.0	20.9	22.0	33.0	−0.19	−0.20	−0.08	−0.17	−0.16	−0.16
3rd codon position	87.1	84.7	91.2	12.9	15.3	8.8	−0.07	−0.07	−0.04	0.19	0.07	−0.16
tRNA genes	79.8	76.8	77.2	20.2	23.2	22.8	0.03	0.02	0.02	−0.11	−0.11	−0.12
lrRNA	84.6	83.2	82.6	15.5	16.8	17.4	0.00	0.05	0.05	−0.30	−0.30	−0.34
srRNA	81.7	79.4	80.5	18.3	20.5	19.5	−0.04	0.01	0.00	−0.30	−0.30	−0.28
Control region	92.4	98.1	92.6	7.7	1.9	7.4	0.11	−0.05	0.11	0.74	1.00	−0.71

Note: *El. fla* indicates *Elodia flavipalpis*, *Ex. sor* indicates *Exorista sorbillans* and *R. goe* indicates *Rutilia goerlingiana*. The A+T and G+C biases of protein-coding genes were calculated by AT-skew = [A−T]/[A+T] and GC-skew = [G−C]/[G+C], respectively.

### Protein-coding Genes and Nucleotide Composition

Thirteen protein-coding genes were identified in the mitogenome of *El. flavipalpis*, with characteristics similar to those of other dipteran species (Table S1). The average A+T content across all protein-coding genes was 79.1%, similar to that of *Ex. sorbillans* (77.7%) and *R. goerlingiana* (76.2%). Table 4 shows the AT-skews and CG-skews of the three codon positions for *El. flavipalpis*, *Ex. sorbillans* and *R. goerlingiana*. In all three of these species, the A+T content of the third codon positions (87.1%, 84.7%, and 91.2%, for *El. flavipalpis*, *Ex. sorbillans*, and *R. goerlingiana,* respectively) was higher than those of the first (71.2%, 70.4%, and 70.1) and second codon positions (79.1%, 78.0%, and 67%). This result is in agreement with studies of other dipteran taxa (Table S1). AT-skew and GC-skew were used to analyse the biases in nucleotide composition. The A content was slightly lower than the T content at all three codon positions, but almost equal over the whole genome.

Except for *CO1* and *ND1*, all of the protein-coding genes have one of the common start codons for mitochondrial DNA, ATG, ATA, or ATT (Table 3). The start codon TCG (Serine) in *CO1* is also found in *Ex. sorbillans*, *R. goerlingiana*, and other Oestroidea species (Table 2). *CO1* commonly uses nonstandard start codons in many other flies [5], [16], [20], [58], [59]. Within Diptera, the start codon TTG (Leucine) in *ND1* of *El. flavipalpis* is same as that in *Dermatobia hominis* (Oestroidea). Of the 13 protein-coding genes, *CO2*, *ND4*, and *ND5* have incomplete stop codons and terminate with only a single thymine. Similar structural features have also been described for the mitogenomes of other dipteran taxa [4], [5]. In addition, *CYTB* terminates with a complete stop codon TAG, whereas TAA or only a single thymine is utilized in some other fly species.

### Other Regions of the Mitogenome

**Figure 2:**
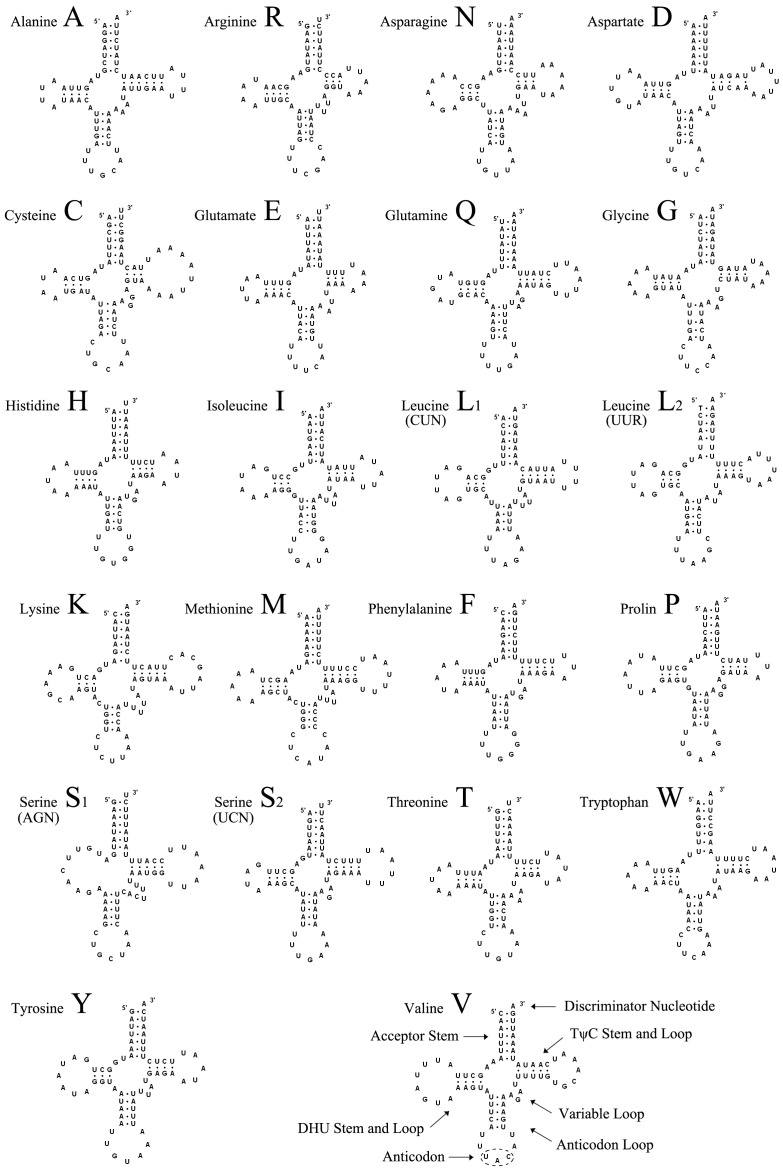
Putative secondary structures of tRNAs found in the mitochondrial genome of *Elodia flavipalpis*. All tRNAs can be folded into the usual clover-leaf secondary structure.

The mitogenome of *El. flavipalpis* bears all of the 22 standard tRNAs found in metazoan mitogenomes (Figure 1, Table 3). The total length of the tRNA genes is 1463 bp, with individual genes ranging from 62 to 71 bp and with A+T contents from 71% (tRNA^Met^) to 92% (tRNA^Glu^). With the exception of tRNA^Ser (AGN)^, all tRNAs possess the typical clover-leaf secondary structure (Figure 2). The DHU-arm of tRNA^Ser (AGN)^ is entirely absent, as observed in other insects [20], [24], [33], [60]. In the secondary structures, the lengths of the amino acid acceptor arms (7 bp), anticodon arms (5 bp), and loops (7 bp) are relatively conserved, while the TψC loop (3–10 bp) is more variable. There are 21 mismatched base pairs in the tRNA genes, 14 of which are weak G–U matches (nine sites in DHU arms, three sites in amino acid acceptor arms, and two sites in anticodon arms). The other seven include U–U (5 bp), C–U (1 bp), and A–A (1 bp).

The lengths of the lrRNA and srRNA genes are 1337 bp and 783 bp, respectively. Both are encoded on the minor strand and the ends of those genes were assumed to be at the boundaries of the flanking genes [61]. As in other dipteran species, the lrRNA gene is flanked by tRNA^Leu (CUN)^ and tRNA^Val^, while the srRNA gene is between tRNA^Val^ and the control region. Their A+T contents were 84.6% for lrRNA and 81.7% for srRNA, which are within the range of other dipteran species (Table S1).

The length of the control region of *El. flavipalpis* is identical to that of *Ex. sorbillans* (105 bp), the shortest among the sequenced dipteran mitogenomes. It has an A+T content of 92.38%, which is lower than that of *Ex. sorbillans* (98.1%) and *R. goerlingiana* (92.6%) but higher than those of most other dipteran species. Owing to its short length, there is no distinct duplicate fragment found in this region. It should be noted that all three of the sequenced tachinid mitogenomes bear a control region that is shorter than those of most known in flies [62], [63].

### Phylogenetic Analysis

**Figure 3:**
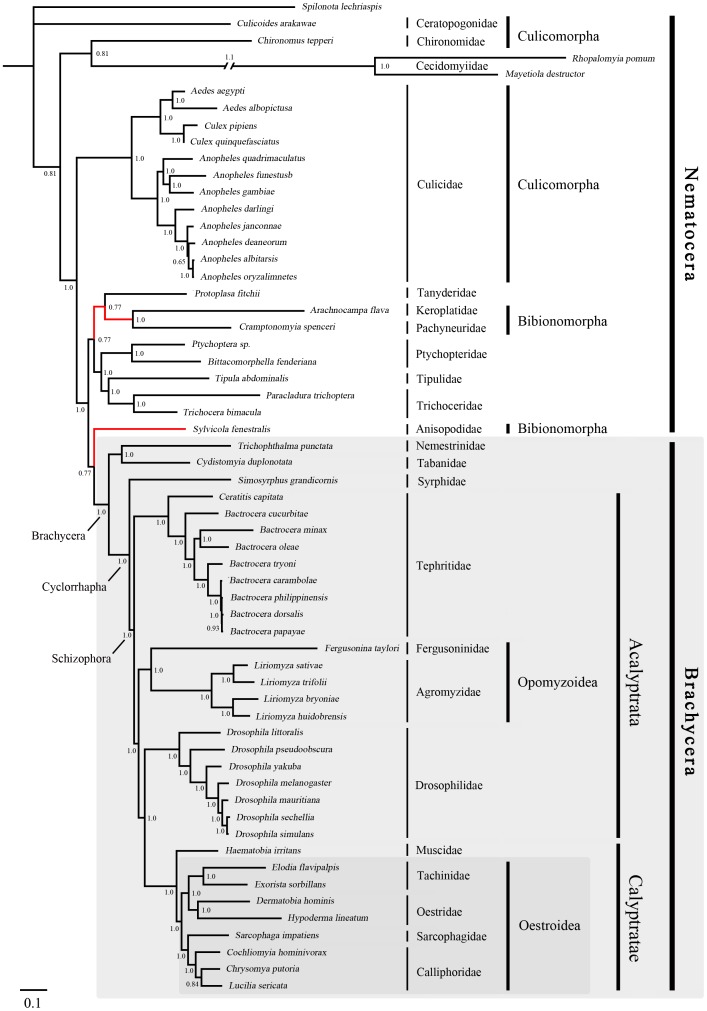
Bayesian tree of Diptera, inferred from a mitochondrial data set comprising 13 protein-coding genes and 2 ribosomal RNA genes. The tree was rooted using the outgroup taxon *Spilonota lechriaspis* (Lepidoptera). Numbers denote posterior probabilities of nodes. The lengths of very long branches have been reduced to aid viewing. The symbol ‘//’ indicates a contracted branch, with the value above giving the length of contraction. Red lines indicate the differences among the four phylogenetic trees.

**Figure 4:**
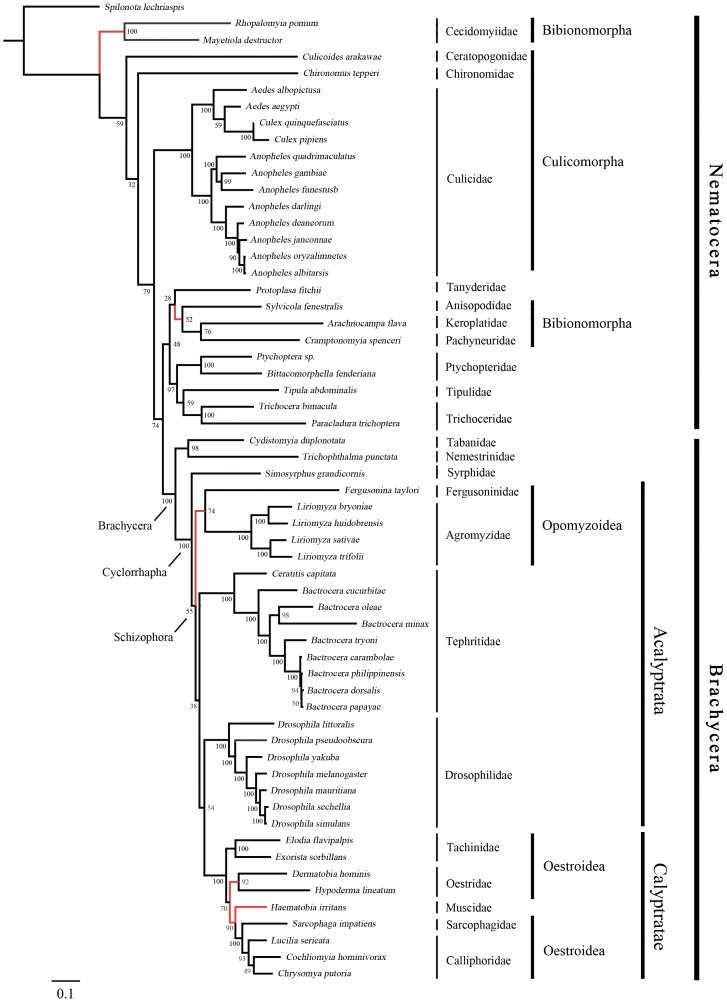
Maximum-likelihood tree of Diptera, inferred from a mitochondrial data set comprising 13 protein-coding genes and 2 ribosomal RNA genes. The tree was rooted using the outgroup taxon *Spilonota lechriaspis* (Lepidoptera). Numbers denote bootstrap values in percentages. Red lines indicate the differences among the four phylogenetic trees.

**Figure 5:**
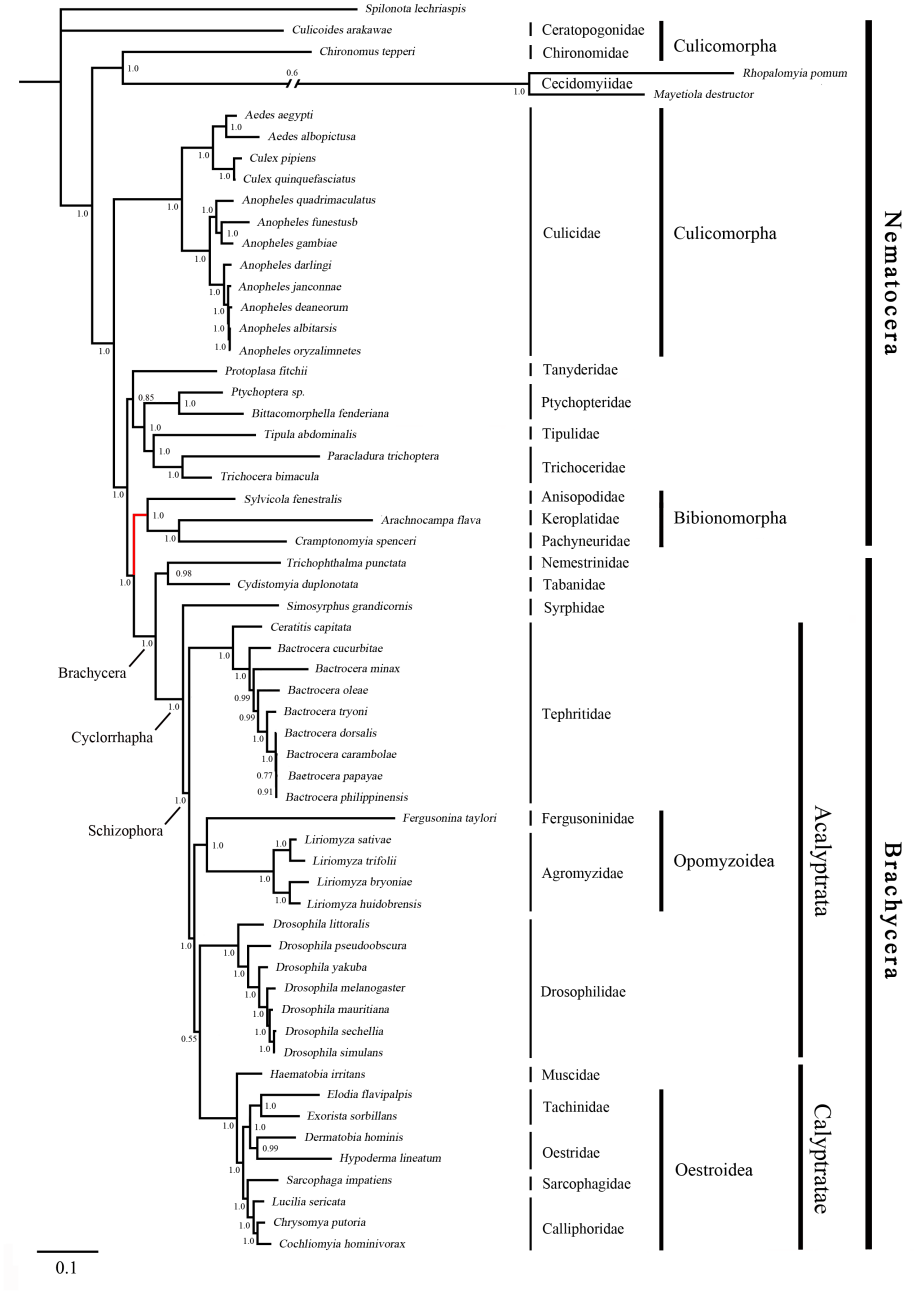
Bayesian tree of Diptera, inferred from a mitochondrial data set comprising 13 protein-coding genes (without third codon sites) and 2 ribosomal RNA genes. The tree was rooted using the outgroup taxon *Spilonota lechriaspis* (Lepidoptera). Numbers denote posterior probabilities of nodes. The lengths of very long branches have been reduced to aid viewing. The symbol ‘//’ indicates a contracted branch, with the value above giving the length of contraction. Red lines indicate the differences among the four phylogenetic trees.

**Figure 6:**
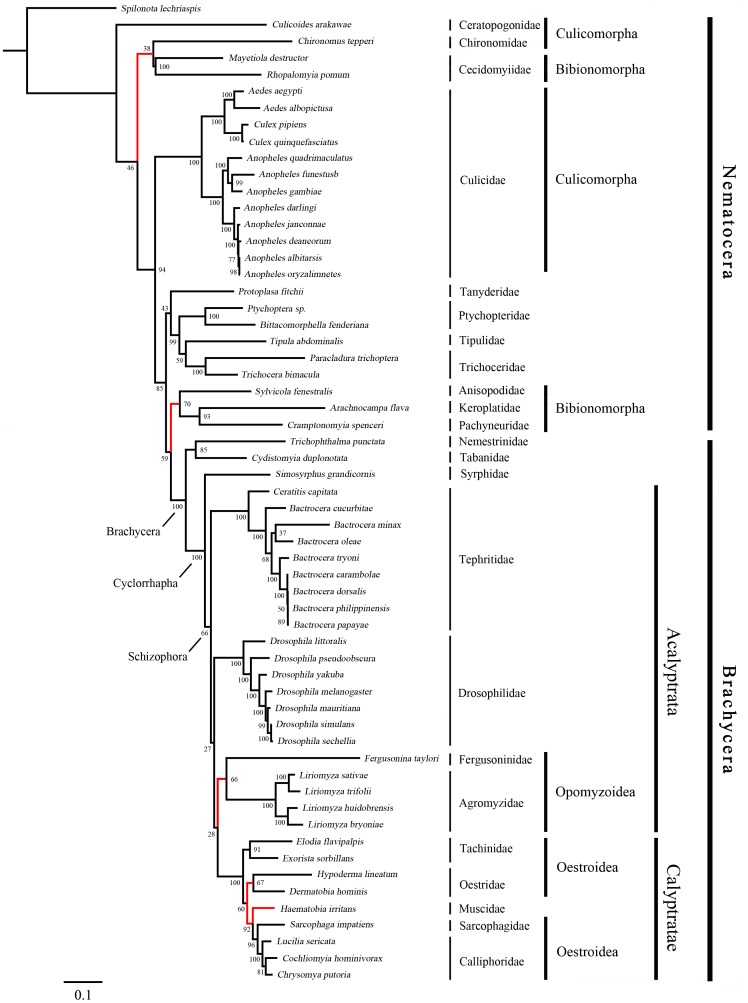
Maximum-likelihood tree of Diptera, inferred from a mitochondrial data set comprising 13 protein-coding genes (without third codon sites) and 2 ribosomal RNA genes. The tree was rooted using the outgroup taxon *Spilonota lechriaspis* (Lepidoptera). Numbers denote bootstrap values in percentages. Red lines indicate the differences among the four phylogenetic trees.

The phylogenies estimated using likelihood and Bayesian approaches were similar for both of the datasets that were analysed (Figures 3, 4, 5 and 6). Various higher-level relationships were consistent across the analyses. The monophyly of Brachycera, Cyclorrhapha, and Calyptratae were consistently supported (posterior probability = 1.00, ML bootstrap = 100), as was the monophyly of Schizophora (posterior probability = 1.00, ML bootstrap = 55, 66). The monophyly of superfamily Oestroidea has been widely accepted and has traditionally received good support from morphological characters [64]–[67]. Here we add support from the Bayesian analysis of mitochondrial DNA sequences that Oestroidea is a sister group of Muscidae (posterior probability = 1.00). However, our ML analyses of both datasets placed the family Muscidae within Oestroidea, although support was not high (bootstrap = 70, 60).

In Oestroidea, all four families are monophyletic, and Calliphoridae+Sarcophagidae was inferred as a sister group to Oestridae+Tachinidae. This result is similar to that obtained in analyses of morphology [34] and of 18S and 16S ribosomal DNAs [35]. The tree topology is broadly similar to that inferred from whole mitogenomes by Nelson et al. [27], except that Oestridae and a subfamily of Calliphoridae (Polleninae) are nested within Tachinidae. However, Nelson et al. [27] focused on the relationships within Calliphoridae, while species from other families of Oestroidea were only used as an outgroup in the analysis.

Our phylogenetic estimate differs from that obtained by Kutty et al. [36] in their analysis of four nuclear and four mitochondrial genes. They inferred the relationships (((Tachinidae, Oestridae), Calliphoridae), Sarcophagidae), with both Calliphoridae and Tachinidae paraphyletic (Oestridae is nested within Tachinidae, as are some calliphorid subfamilies). In contrast, Wiegmann et al. [28], considered Tachinidae to be more closely related to Calliphoridae, and as a sister taxon to Oestridae and Sarcophagidae. Owing to the morphological similarities shared by these four families, it is difficult to distinguish among these phylogenetic hypotheses using morphological data. The disparities among the molecular estimates are probably due to differences in the taxa sampled and the data being analysed. Kutty et al. [36] and Wiegmann et al. [28] used both nuclear and mitochondrial DNA sequences, but most were only partial sequences. Moreover, most of the Oestroidea species analysed by Wiegmann et al. [28] were represented by only two or three genes. Given that our analysis involved a larger and more complete data set, we believe that our results are more strongly supported.

The primary difference among the four phylogenetic trees here is in the placement of the superfamily Opomyzoidae, which consists of the families Furgusoninidae and Agromyzidae. It is closer to Drosophoridae in the Bayesian analysis (posterior probability = 1.00), but its position is unstable in the ML analysis (bootstrap = 55, 27). A similar result was obtained by Wiegmann et al. [28]. Another difference is seen in the placement of Bibionomorpha (Nematocera), which is the closest group to Brachycera in both of the trees inferred without third codon positions, but is unstable in the tree inferred from all three gene positions. It is even non-monophyletic in the Bayesian analysis, hinting at the possible negative phylogenetic effects of including third codon sites. The placement of Cecidomyiidae is problematic, which is also indicated by the long branch leading to this group. The two Cecidomyiidae species have undergone substantial reduction in mitogenome size, which results in their apparent distinctiveness and causes problems for sequence alignment. With additional sampling in Cecidomyiidae, future studies will be better equipped to reconstruct the molecular evolution of these mitochondrial genomes.

### Estimates of Divergence Times

**Figure 7:**
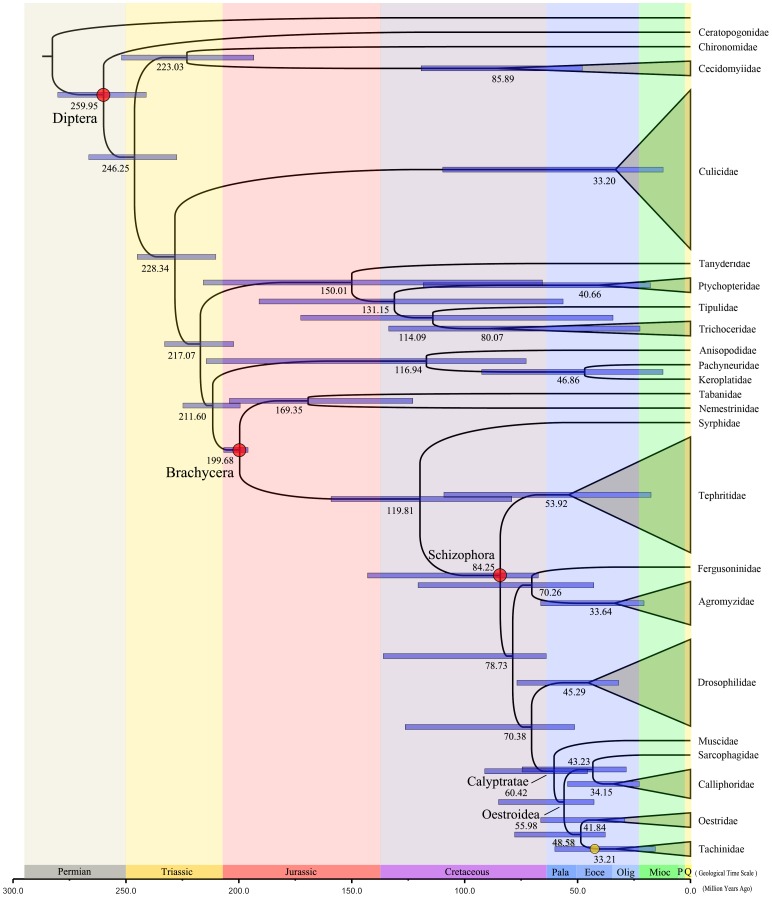
Evolutionary timescale for Diptera inferred from a mitochondrial data set comprising 13 protein-coding genes and 2 ribosomal RNA genes. Numbers at nodes indicate mean estimated divergence times (in mya) and node bars indicate 95% credibility intervals. Red circles indicate the three nodes used for calibration. The yellow circle indicates the hypothesised origin of tachinid flies. In the geological time scale: Pala indicates Palaeocene; Eoce indicates Eocene; Oligo indicates Oligocene; Mioc indicates Miocene; P indicates Pliocene; Q indicates Quaternary.

We used a Bayesian relaxed clock to estimate the evolutionary timescale of Brachycera (Figure 7). Our analysis suggests that the last common ancestor of extant Brachycera existed in the early Jurassic (∼199 mya) (95% credibility interval: 195.9–206.7 mya). The schizophoran radiation took place during the late Cretaceous (∼84 mya), and the clade Calyptratae containing Oestroidea and Muscidae (nested in Acalyptrata) split in the early Eocene (∼60 mya) (95% credibility interval: 45.6–91.1 mya). These results are consistent with evidence from a number of amber specimens from Acalyptrata and Calyptratae [68]. Subsequently, Oestroidea divided into two groups in the middle Palaeocene (∼56 mya) (95% credibility interval (CI): 42.7–85.0 mya). The split of Tachinidae with Oestridae is estimated to have occurred in the early Eocene (∼48 mya) (95% CI: 37.8–77.9 mya), with this clade becoming the most speciose in Brachycera. Finally, the two tachinid flies *El. flavipalpis* and *Ex. sorbillans* are estimated to have separated in the early Oligocene (∼33 mya) (95% CI: 15.5–60.1 mya).

Our estimates suggest that the most recent common ancestor of tachinid flies existed between 33 and 48 mya. The two tachinid samples used in this study are from the same subfamily Exoristinae, but are in separate tribes Goniini (*El. flavipalpis*) and Exoristini (*Ex. sorbillans*). Among the four subfamilies of Tachinidae, Exoristinae was probably the latest to emerge [69]–[71]. Therefore, the time of origin of tachinid flies should be in the earlier half of the time interval described above. We speculate that the most recent common ancestor of tachinid flies existed in the middle Eocene (∼42–48 mya). Tachinid flies have a worldwide distribution, but are mainly found in Palaearctic and Nearctic regions. For a globally distributed family with significant differences in distribution between northern and southern continents, the relevant split is between the supercontinents Laurasia and Gondwana in the mid-Mesozoic. Our estimates for the origin of Tachinidae is much more recent than this. However, given that most tachinid species are distributed throughout the Holarctic Region, we suggest that the evolutionary history of tachinid flies is probably tied to the split of Laurasia in the Eocene rather than that between Laurasia and Gondwana.

We have shown that the availability of additional mitogenomes can make a valuable contribution to our understanding of the phylogeny and divergence times of Diptera. Our study serves as a useful primer for the evolution of tachinid flies, but the accuracy of divergence-time estimates can be improved by denser sampling of tachinid mitogenomes, a greater number of fossil calibrations, and combination with nuclear genes or morphological data. In addition, we suggest that the stable gene order among brachyceran species might be due to their comparatively short evolutionary timeframe.

## Supporting Information

Table S1 Nucleotide composition of all available mitogenome sequences from Diptera.
(DOC)
