# Supplementary material for: The Mitochondrial Genome of Elodia flavipalpis Aldrich (Diptera: Tachinidae) and the Evolutionary Timescale of Tachinid Flies
Source: PLoS One. 2013 Apr 23;8(4):e61814. doi: 10.1371/journal.pone.0061814 (PMC3634017; doi:10.1371/journal.pone.0061814)
Supplement: Table S1 — Nucleotide composition of all available mitogenome sequences from Diptera. (DOC) [file pone.0061814.s001.doc]

**Table S1**. Nucleotide composition of all available mitogenome sequences from Diptera.

| Species | Length  (bp) | AT% | Protein |  | tRNA |  | rRNA |  | Control region | |
| --- | --- | --- | --- | --- | --- | --- | --- | --- | --- | --- |
|  |  |  | Codons /  Amino Acid | AT% | Codons /  Number | AT% | Codons | AT% | Length | AT% |
| *Elodia flavipalpis* | 14932 | 79.9 | 11193 / 3720 | 79.1 | 1463 / 22 | 79.8 | 2120 | 83.5 | 105 | 92.4 |
| *Exorista sorbillans* | 14960 | 78.4 | 11170 / 3712 | 77.7 | 1471 / 22 | 76.8 | 2117 | 81.8 | 105 | 98.1 |
| *Rutilia goerlingiana* | 15331 | 77.7 | 11158 / 3711 | 76.2 | 1451 / 22 | 77.2 | 2101 | 81.8 | 568 | 92.6 |
| *Dermatobia hominis* | 16360 | 77.8 | 11190 / 3719 | 75.3 | 1524 / 23 | 77.5 | 2112 | 81.4 | 1545 | 91.3 |
| *Hypoderma lineatum* | 16354 | 77.8 | 11181 / 3715 | 75.9 | 1453 / 22 | 77.7 | 2101 | 80.5 | 1493 | 87.5 |
| *Chrysomya putoria* | 15837 | 76.7 | 11185 / 3718 | 74.9 | 1537 / 23 | 76.1 | 2114 | 80.0 | 1008 | 88.6 |
| *Cochliomyia hominivorax* | 16022 | 76.9 | 11190 / 3719 | 74.8 | 1470 / 22 | 76.6 | 2110 | 79.8 | 1117 | 90.8 |
| *Lucilia sericata* | 15939 | 77.6 | 11183 / 3718 | 76.0 | 1468 / 22 | 76.8 | 2112 | 80.0 | 1127 | 90.2 |
| *Sarcophaga impatiens* | 15169 | 74.8 | 11185 / 3718 | 73.1 | 1469 / 22 | 76.4 | 2113 | 79.5 | 359 | 88.3 |
| *Haematobia irritans* | 16078 | 79.1 | 11183 / 3718 | 77.7 | 1465 / 22 | 77.0 | 2105 | 81.3 | 1259 | 89.4 |
| *Drosophila littoralis* | 16017 | 76.2 | 11175 / 3716 | 73.9 | 1478 / 22 | 76.2 | 2109 | 80.8 | 1023 | 90.1 |
| *Drosophila sechellia* | 14950 | 77.5 | 11177 / 3715 | 76.7 | 1468 / 22 | 76.7 | 2108 | 81.6 | - | - |
| *Drosophila simulans* | 14927 | 77.9 | 11174 / 3715 | 77.0 | 1466 / 22 | 77.0 | 2110 | 81.9 | - | - |
| *Drosophila mauritiana* | 14964 | 77.7 | 11173 / 3715 | 76.8 | 1466 / 22 | 76.1 | 2109 | 81.9 | - | - |
| *Drosophila melanogaster* | 19517 | 82.2 | 11181 / 3716 | 77.2 | 1457 / 22 | 77.1 | 2111 | 81.9 | 4601 | 95.6 |
| *Drosophila yakuba* | 16019 | 78.6 | 11212 / 3727 | 76.7 | 1465 / 22 | 76.6 | 2115 | 81.9 | 1077 | 92.9 |
| *Drosophila pseudoobscura* | 14914 | 77.1 | 11180 / 3715 | 76.2 | 1449 / 22 | 76.1 | 2093 | 81.3 | - | - |
| *Liriomyza sativae* | 15551 | 77.5 | 11193 / 3720 | 75.7 | 1465 / 22 | 77.0 | 2111 | 82.2 | 741 | 93.0 |
| *Liriomyza trifolii* | 16141 | 78.0 | 11177 / 3720 | 75.4 | 1671 / 24 | 79.1 | 2107 | 82.6 | 1338 | 93.3 |
| *Liriomyza bryoniae* | 16183 | 79.3 | 11201 / 3723 | 76.7 | 1473 / 22 | 78.7 | 2111 | 82.4 | 1354 | 95.5 |
| *Liriomyza huidobrensis* | 16236 | 78.3 | 11198 / 3722 | 73.7 | 1466 / 22 | 77.4 | 2109 | 82.2 | 1416 | 93.0 |
| *Fergusonina taylori* | 16000 | 78.2 | 11166 / 3714 | 76.1 | 1472 / 22 | 79.3 | 2086 | 82.4 | 1057 | 88.9 |
| *Ceratitis capitata* | 15980 | 77.5 | 11183 / 3716 | 75.6 | 1472 / 22 | 76.8 | 2123 | 80.2 | 1004 | 91.1 |
| *Bactrocera carambolae* | 15915 | 73.6 | 11190 / 3719 | 71.1 | 1466 / 22 | 75.1 | 2113 | 77.5 | 950 | 87.9 |
| *Bactrocera dorsalis* | 15915 | 73.6 | 11185 / 3719 | 71.2 | 1467 / 22 | 76.1 | 2123 | 77.8 | 949 | 88.1 |
| *Bactrocera minax* | 16043 | 67.3 | 11183 / 3717 | 64.3 | 1466 / 22 | 72.3 | 2115 | 73.7 | 1140 | 77.6 |
| *Bactrocera oleae* | 15815 | 72.6 | 11189 / 3718 | 70.2 | 1466 / 22 | 74.8 | 2116 | 77.1 | 949 | 86.9 |
| *Bactrocera papayae* | 15915 | 73.5 | 11190 / 3719 | 71.0 | 1463 / 22 | 75.0 | 2114 | 77.7 | 950 | 88.2 |
| *Bactrocera philippinensis* | 15915 | 73.6 | 11192 / 3719 | 71.2 | 1466 / 22 | 75.3 | 2114 | 77.7 | 949 | 88.2 |
| *Bactrocera tryoni* | 15925 | 72.5 | 11187 / 3719 | 69.6 | 1467 / 22 | 75.0 | 2115 | 77.7 | 951 | 87.0 |
| *Bactrocera cucurbitae* | 15825 | 72.9 | 11190 / 3719 | 70.7 | 1467 / 22 | 75.1 | 2110 | 77.8 | 946 | 82.4 |
| *Simosyrphus grandicornis* | 16141 | 80.9 | 11213 / 3726 | 78.9 | 1480 / 22 | 80.4 | 2143 | 84.6 | 1129 | 91.8 |
| *Trichophthalma punctata* | 16396 | 74.0 | 11207 / 3726 | 72.0 | 1459 / 22 | 74.9 | 2101 | 78.2 | 2379 | 79.9 |
| *Cydistomyia duplonotata* | 16247 | 77.9 | 11192 / 3722 | 75.6 | 1538 / 23 | 77.8 | 2130 | 81.0 | 1376 | 92.6 |
| *Rhopalomyia pomum* | 14503 | 85.2 | 10879 / 3615 | 83.5 | 1160 / 22 | 90.4 | 1995 | 88.7 | 363 | 94.2 |
| *Mayetiola destructor* | 14759 | 84.1 | 10927 / 3629 | 82.2 | 1219 / 22 | 90.7 | 2048 | 88.1 | 604 | 90.9 |
| *Culicoides arakawae* | 18132 | 77.3 | 11231 / 3734 | 73.9 | 1434 / 22 | 77.9 | 2095 | 83.9 | 1435 | 90.5 |
| *Aedes aegypti* | 16655 | 79.0 | 11191 / 3720 | 75.9 | 1488 / 22 | 79.3 | 2125 | 82.6 | 1709 | 93.5 |
| *Aedes albopictus*^a^ | 16665 | 79.6 | 11225 / 3730 | 77.0 | 1485 / 22 | 79.5 | 2137 | 82.2 | 1772 | 91.6 |
| *Anopheles darlingi* | 15386 | 78.2 | 11235 / 3735 | 76.7 | 1489 / 22 | 78.5 | 2124 | 81.6 | 554 | 93.7 |
| *Anopheles funestus^b^* | - | - | - / - | - | 1476 / 22 | 78.7 | - | - | 516 | 93.8 |
| *Anopheles gambiae* | 15363 | 77.5 | 11230 / 3733 | 76.0 | 1478 / 22 | 77.9 | 2125 | 81.4 | 519 | 94.3 |
| *Anopheles quadrimaculatus* | 15455 | 77.4 | 11220 / 3729 | 75.5 | 1473 / 22 | 78.7 | 2115 | 81.6 | 625 | 93.5 |
| *Anopheles albitarsis* | 15413 | 77.7 | 11216 / 3731 | 76.1 | 1477 / 22 | 78.7 | 2119 | 81.2 | 575 | 93.2 |
| *Anopheles deaneorum* | 15424 | 77.8 | 11216 / 3731 | 76.2 | 1476 / 22 | 78.3 | 2121 | 81.4 | 581 | 92.3 |
| *Anopheles janconnae* | 15425 | 77.6 | 11216 / 3731 | 76.0 | 1480 / 22 | 78.4 | 2120 | 81.5 | 575 | 92.4 |
| *Anopheles oryzalimnetes* | 15422 | 77.8 | 11216 / 3731 | 76.2 | 1487 / 22 | 78.6 | 2120 | 81.2 | 581 | 94.1 |
| *Culex pipiens* | 14856 | 77.7 | 11216 / 3729 | 76.5 | 1475 / 22 | 79.0 | 2118 | 82.2 | - | - |
| *Culex quinquefasciatus* | 15587 | 78.0 | 11216 / 3729 | 76.3 | 1479 / 22 | 78.7 | 2137 | 82.3 | 704 | 88.5 |
| *Chironomus tepperi* | 15652 | 76.9 | 11229 / 3730 | 74.4 | 1493 / 22 | 79.0 | 2192 | 83.7 | 500 | 93.0 |
| *Trichocera bimacula* | 16140 | 75.2 | 11230 / 3731 | 72.6 | 1470 / 22 | 75.3 | 2116 | 80.2 | 1049 | 89.1 |
| *Paracladura trichoptera* | 16143 | 78.3 | 11206 / 3723 | 76.2 | 1462 / 22 | 81.4 | 2177 | 80.9 | 724 | 84.8 |
| *Sylvicola fenestralis* | 16234 | 76.4 | 11237 / 3734 | 73.9 | 1473 / 22 | 76.9 | 2133 | 81.0 | 1232 | 86.0 |
| *Bittacomorphella fenderiana* | 15609 | 76.9 | 11222 / 3729 | 75.3 | 1474 / 22 | 78.9 | 2201 | 79.7 | 723 | 87.7 |
| *Ptychoptera sp.* | 15214 | 75.9 | 11212 / 3725 | 74.5 | 1475 / 22 | 77.0 | 2123 | 79.0 | 370 | 94.0 |
| *Protoplasa fitchii* | 16154 | 77.1 | 11192 / 3719 | 74.6 | 1478 / 22 | 77.1 | 2137 | 80.9 | 1255 | 92.1 |
| *Cramptonomyia spenceri* | 16274 | 76.1 | 11223 / 3729 | 72.8 | 1486 / 22 | 79.9 | 2138 | 81.7 | 1069 | 90.6 |
| *Arachnocampa flava* | 16923 | 82.0 | 11202 / 3722 | 79.0 | 1506 / 22 | 84.5 | 2103 | 84.9 | 1841 | 93.3 |
| *Tipula abdominalis* | 14566 | 74.7 | 11209 / 3725 | 73.6 | - / 22 | - | - | - | - | - |

Note ‘-‘ not available (unknown or incomplete data).
